# A Combination of Chest Radiography and Estimated Plasma Volume May Predict In-Hospital Mortality in Acute Heart Failure

**DOI:** 10.3389/fcvm.2021.752915

**Published:** 2022-01-11

**Authors:** Masatake Kobayashi, Amine Douair, Stefano Coiro, Gaetan Giacomin, Adrien Bassand, Déborah Jaeger, Kevin Duarte, Olivier Huttin, Faiez Zannad, Patrick Rossignol, Tahar Chouihed, Nicolas Girerd

**Affiliations:** ^1^Université de Lorraine, Centre d'Investigations Cliniques Plurithématique 1433, INSERM 1116, Nancy, France; ^2^F-CRIN INI-CRCT Cardiovascular and Renal Clinical Trialists Network, Nancy, France; ^3^CHRU Nancy, F-CRIN INI-CRCT, Nancy, France; ^4^Department of Cardiology, Tokyo Medical University, Tokyo, Japan; ^5^Emergency Department, University Hospital of Nancy, Nancy, France; ^6^Division of Cardiology, University of Perugia, Perugia, Italy

**Keywords:** acute heart failure, chest x ray, estimated plasma volume, congestion, emergency and critical care

## Abstract

**Background:** Patients with heart failure (HF) often display dyspnea associated with pulmonary congestion, along with intravascular congestion, both may result in urgent hospitalization and subsequent death. A combination of radiographic pulmonary congestion and plasma volume might screen patients with a high risk of in-hospital mortality in the emergency department (ED).

**Methods:** In the pathway of dyspneic patients in emergency (PARADISE) cohort, patients admitted for acute HF were stratified into 4 groups based on high or low congestion score index (CSI, ranging from 0 to 3, high value indicating severe congestion) and estimated plasma volume status (ePVS) calculated from hemoglobin/hematocrit.

**Results:** In a total of 252 patients (mean age, 81.9 years; male, 46.8%), CSI and ePVS were not correlated (Spearman rho <0 .10, *p* > 0.10). High CSI/high ePVS was associated with poorer renal function, but clinical congestion markers (i.e., natriuretic peptide) were comparable across CSI/ePVS categories. High CSI/high ePVS was associated with a four-fold higher risk of in-hospital mortality (adjusted-OR, 95%CI = 4.20, 1.10-19.67) compared with low CSI/low ePVS, whereas neither high CSI nor ePVS alone was associated with poor prognosis (all-*p*-value > 0.10; P_interaction_ = 0.03). High CSI/high ePVS improved a routine risk model (i.e., natriuretic peptide and lactate)(NRI = 46.9%, *p* = 0.02), resulting in high prediction of risk of in-hospital mortality (AUC = 0.85, 0.82-0.89).

**Conclusion:** In patients hospitalized for acute HF with relatively old age and comorbidity burdens, a combination of CSI and ePVS was associated with a risk of in-hospital death, and improved prognostic performance on top of a conventional risk model.

## Introduction

Congestion in heart failure (HF) is defined as a high left ventricular filling pressure associated with signs and symptoms of HF such as dyspnea and edema (1–3). Patients with acute HF (AHF) often require urgent emergency department (ED) admissions due to acute dyspnea, which may be related to both pulmonary and intravascular congestion (2–5). Once treatment of congestion has been initiated in the ED and their effects have been assessed, clinical decisions are made about subsequent hospital management. However, whether the severity of congestion in the ED is associated with a higher risk of in-hospital mortality has not been well documented (6–8). Assessment of integrating pulmonary and intravascular congestion might provide better patient risk stratification.

The congestion score index (CSI) is a semi-quantitative method for assessing pulmonary congestion on chest radiography (CXR) (9–11). Plasma volume (PV), on the other hand, expresses the intravascular portion of the extracellular fluid volume, and is estimated from hemoglobin/hematocrit levels (12–14). Its estimation (estimated plasma volume status, ePVS) has been associated with left ventricular (LV) filling pressure (i.e., E/e' and invasively-measured LV end-diastolic pressure) (15) and with pulmonary congestion (16). Recent published reports showed that ePVS and CSI were associated with clinical outcomes in several HF settings (10–14). Accordingly, our group reported that increased both ePVS and CSI of AHF patients were associated with a high risk of 90-day post-discharge outcomes (9). We hypothesized that the combination of elevated CSI and ePVS could potentially identify patients with markedly increased congestion in the ED, and provide important risk-stratification for in-hospital management.

The main objectives of the present study are to (i) assess the interplay between CSI and ePVS in patients admitted for AHF in the ED and (ii) explore the association of CSI and/or ePVS with in-hospital mortality.

## Methods

### Study Population

The Pathway of dyspneic patients in Emergency (PARADISE) cohort is a retrospective cohort study including consecutive acute dyspneic patients aged 18 years or older who admitted for the ED of the Nancy University Hospital (France) between January 1, 2015 and December 31, 2015 as previously reported (14, 17, 18). These patients were systematically reviewed and selected by investigators using the hospital's electronic ED charts (RESURGENCES). Then, two medical physicians independently labeled patients with AHF per ESC guidelines (19) based on medical admission examination records (e.g., echocardiogram, natriuretic peptide level and patient response to diuretic/bronchodilators therapy). Only patients with AHF diagnosis were included in the current analysis. Importantly, the aforementioned physicians had no access to CSI quantification on CXR, which was performed after this patient selection.

Among 1,589 patients included in the PARADISE cohort, 252 AHF patients with available data on CXR and hemoglobin/hematocrit were studied. Those with length of hospital stay > 30 days were excluded since they likely had either atypical degrees of congestion or non-congestion related problem (Supplementary Figure 1). Demographic parameters, medical history, physical examination, laboratory findings and treatment received in the ED were retrieved from the patient electronic records. The PARADISE cohort was approved by the Commission Nationale de l'Informatique et des Libertés (CNIL) (Number R2016-08) and registered on “Clinicaltrials.gov” (NCT02800122).

### Radiographic Congestion Score Index

**Figure 1:**
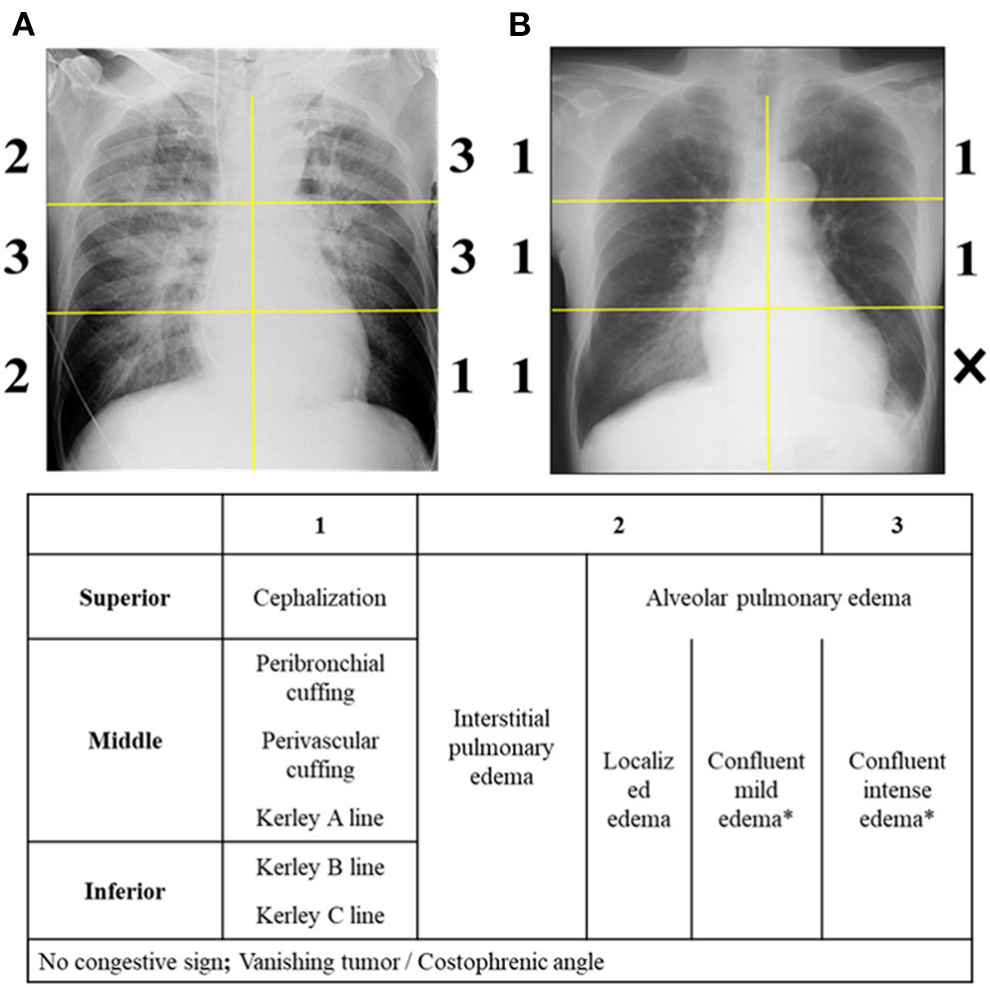
Radiographic congestion score index. The scoring is performed on six lung fields. **(A,B)** Provide examples of grades 0-3. ^*^Confluent edema was regarded as intense edema when the density in an area of the divided lung field was visually similar to that of cardiac silhouette.

CSI is assessed to quantify the severity of pulmonary congestion on CXR as previously described (9, 10). After dividing the lung field into six equal zones, each area was assessed as follows: Score 0, no congestion sign; Score 1, cephalization (superior area), perihilar haze or perivascular/peribronchial cuffing or Kerley's A lines (middle area), Kerley's B or C lines (inferior area); Score 2, interstitial or localized/mild alveolar pulmonary edema; Score 3, intense alveolar pulmonary edema (Figure 1). To enhance the reproducibility of confluent edema severity, a portion of the lung fields which was visually similar than the cardiac silhouette was considered as an intense edema, whereas the field with weaker density was considered as a mildly intense edema. Lung areas were not scored when more than one third of the divided lung fields were occupied by pleural effusion (including vanishing tumor), atelectasis or cardiac silhouette. CSI was calculated as the sum of the scores in each zone divided by the number of available zones. An examiner also assessed the presence of pneumonia, pleural effusion, and the difficulty in assessing CSI.

CXR was analyzed by a single emergency physician (AD), blinded to clinical data and outcome, with no prior training in CSI. After a short training (~3 h) using a 20-patient sample with a radiographic CSI expert (MK), when blinded to clinical data and outcome, intra-class correlation coefficients showed good reproducibility (intra-observer agreement [95%CI], 0.85 [0.71-0.93]; inter-observer agreement [95%CI] with MK, 0.81 [0.64–0.90]).

### Estimated Plasma Volume Status

ePVS was calculated from the Strauss-derived Duarte formula using hematocrit and hemoglobin (12, 20) as follows: ePVS (ml/g) = 100 × (1-hematocrit)/hemoglobin in g/dl.

### Statistical Analysis

Patients were divided into four groups according to sex-specific median ePVS (4.64 ml/g in male and 5.03 ml/g in female) and median CSI (2.20); low CSI/low ePVS, high CSI/low ePVS, low CSI/high ePVS, and high CSI/high ePVS. Categorical variables are presented as frequencies (percentages) and continuous variables as means ± standard deviation or median (25th and 75th percentiles), according to variable distribution. Comparisons of demographic, clinical and biological parameters across CSI/ePVS categories were analyzed using variances, Kruskal-Wallis test and χ^2^ test, as appropriate. Inter-observer and intra-observer agreements of CSI were assessed with intra-class correlation coefficients.

Logistic regression analysis was used to study the associations of CSI/ePVS categories with in-hospital mortality. The relevant covariates as previously shown were followings (7, 9): age, sex, prior HF hospitalization, a previous use of diuretics therapy, continuous positive airway pressure (CPAP), blood urea nitrogen (BUN), estimated glomerular filtration rate [eGFR, as calculated from the Chronic Kidney Disease Epidemiology Collaboration formula (21)], brain natriuretic peptide (BNP) and lactate. An interaction test was performed to determine the likelihood of interaction between CSI and ePVS on in-hospital mortality. Interactions between CSI/ePVS categories and baseline clinical presentations on the outcome were also tested. In addition, time-to-event comparisons were analyzed using log rank test and Cox proportional hazards models. Survival probabilities were estimated using the Kaplan-Meier method and plotted as survival curves with CSI/ePVS categories.

Furthermore, net reclassification improvement (NRI) was used to assess the increase in discriminative value of the addition of high CSI/high ePVS on the top of the aforementioned covariates including clinical model (i.e., age, sex, prior HF admission, diuretics, and CPAP) plus biological model (i.e., BUN, eGFR, BNP and lactate at admission). The continuous NRI method was implemented in the survIDINRI package of the R software (22).

A *p*-value < 0.05 was considered significant. No imputation was performed. Statistical analyses were performed using the R statistical software (3.4.0).

## Results

Approximately a half of the 252 included patients were male (46.8%), mean age was 81.9 ± 9.8 years, mean body mass index was 26.7 ± 6.4 kg/m^2^ and 22.2% had a prior admission for HF. Median BNP was 516 (271-1,001) pg/ml (Table 1). Median (IQR) CSI and ePVS were 2.2 (1.8-2.5) and 4.9 (4.0-5.9) ml/g, respectively. There was not significant correlation between them (Spearman rho <0.10, *p* = 0.44).

**Table 1:** Baseline characteristics according to CSI/ePVS subsets.

	**Overall (*N* = 252)**	**Low CSI** **Low ePVS** **(*N* = 68)**	**High CSI** **Low ePVS** **(*N* = 58)**	**Low CSI** **High ePVS** **(*N* = 57)**	**High CSI** **High ePVS** **(*N* = 69)**	***P*-value**
Age, yrs	81.9 ± 9.8	81.1 ± 9.7	81.3 ± 10.6	81.7 ± 8.5	83.5 ± 10.4	0.47
Male, *N* (%)	118 (46.8 %)	28 (41.2 %)	31 (53.4 %)	28 (49.1 %)	31 (44.9 %)	0.55
Body mass index, kg/m^2^	26.7 ± 6.4	26.9 ± 6.6	27.6 ± 7.4	25.6 ± 5.6	26.8 ± 5.8	0.37
**Medical history**, ***N*** **(%)**						
Hypertension	183 (72.6 %)	46 (67.6 %)	39 (67.2 %)	44 (77.2 %)	54 (78.3 %)	0.33
Diabetes mellitus	94 (37.3 %)	19 (27.9 %)	26 (44.8 %)	22 (38.6 %)	27 (39.1 %)	0.25
Coronary artery disease	52 (20.6 %)	14 (20.6 %)	11 (19.0 %)	15 (26.3 %)	12 (17.4 %)	0.64
Atrial fibrillation	109 (43.3 %)	26 (38.2 %)	26 (44.8 %)	22 (38.6 %)	35 (50.7 %)	0.42
COPD	94 (37.3 %)	26 (38.2 %)	23 (39.7 %)	21 (36.8 %)	24 (34.8 %)	0.95
Prior HF hospitalization	56 (22.2 %)	14 (20.6 %)	11 (19.0 %)	11 (19.3 %)	20 (29.0 %)	0.46
**Medications at admission**, ***N*** **(%)**						
ACEi/ARB	105 (43.0 %)	22 (33.3 %)	27 (48.2 %)	27 (49.1 %)	29 (43.3 %)	0.26
Beta-blocker	91 (37.3 %)	24 (36.4 %)	21 (37.5 %)	21 (38.2 %)	25 (37.3 %)	0.99
Spironolactone	16 (6.6 %)	6 (9.1 %)	3 (5.4 %)	1 (1.8 %)	6 (9.0 %)	0.32
Diuretics	102 (41.8 %)	19 (28.8 %)	25 (44.6 %)	21 (38.2 %)	37 (55.2 %)	**0.02**
**Intensive treatment**, ***N*** **(%)**						
Vasodilator	52 (20.6 %)	14 (20.6 %)	19 (32.8 %)	8 (14.0 %)	11 (15.9 %)	0.054
CPAP	87 (34.5 %)	18 (26.5 %)	29 (50.0 %)	17 (29.8 %)	23 (33.3 %)	**0.03**
**Physical examination**						
Leg edema, *N* (%)	141 (56.0 %)	35 (51.5 %)	29 (50.0 %)	31 (54.4 %)	46 (66.7 %)	0.20
Rales, *N* (%)	149 (61.6 %)	36 (54.5 %)	36 (65.5 %)	35 (63.6 %)	42 (63.6 %)	0.58
Systolic BP, mmHg	138.7 ± 28.6	138.6 ± 29.5	142.4 ± 30.2	139.6 ± 27.6	135.0 ± 27.3	0.53
Heart rate, bpm	92.6 ± 22.0	97.1 ± 19.9	90.6 ± 23.4	89.2 ± 19.6	92.6 ± 24.1	0.20
Respiratory rate, /min	27.2 ± 7.9	28.1 ± 7.5	28.1 ± 8.9	26.7 ± 7.3	26.1 ± 7.8	0.45
Body temperature, °C	37.1 ± 1.1	37.2 ± 1.1	37.3 ± 1.3	37.1 ± 1.2	37.0 ± 1.1	0.70
**Chest radiography**						
Congestion score index	2.2 (1.8-2.5)	2.0 (1.4-2.0)	2.6 (2.4-2.8)	1.8 (1.6-2.0)	2.5 (2.3-2.7)	**<0.001**
Pleural effusion, *N* (%)	8 (14.3 %)	7 (10.3 %)	7 (12.1 %)	8 (14.3 %)	12 (17.4 %)	0.65
**Biological findings**						
Hemoglobin, g/dl	12.4 ± 2.0	14.1 ± 1.2	13.8 ± 1.1	10.8 ± 1.1	10.8 ± 1.2	**<0.001**
Hematocrit, %	39.4 ± 6.0	44.1 ± 3.9	44.2 ± 3.7	34.7 ± 3.5	34.8 ± 3.8	**<0.001**
White blood count, 10^3^/μl	12.4 (11.0-13.9)	11.9 (8.8-14.5)	13.7 (9.0-15.6)	10.9 (7.3-15.4)	12.9 (8.8-17.4)	0.20
CRP, mg/dl	5.0 (1.8-11.9)	5.4 (2.1-11.3)	3.0 (1.3-9.4)	5.0 (2.0-16.2)	6.9 (1.9-13.6)	0.11
Sodium, mmol/l	1.49 ± 1.27	136.6 ± 5.1	137.1 ± 3.9	137.2 ± 4.8	137.2 ± 5.3	0.88
Potassium, mmol/l	4.2 ± 0.7	4.2 ± 0.5	4.1 ± 0.6	4.1 ± 0.7	4.5 ± 0.8	**0.005**
Blood urea nitrogen, mg/dl	30.6 ± 20.3	25.8 ± 16.6	25.6 ± 10.8	33.0 ± 18.1	37.6 ± 28.0	**<0.001**
eGFR, ml/min/1.73m^2^	66.7 ± 31.7	74.8 ± 31.7	69.5 ± 29.8	65.0 ± 33.0	57.4 ± 30.2	**0.006**
BNP, pg/ml	516 (271-1001)	403 (196-754)	603 (291-1114)	550 (335-1045)	602 (340-998)	0.07
ePVS, ml/g	4.9 (4.0-5.9)	4.0 (3.6-4.4)	4.0 (3.8-4.4)	5.9 (5.3-6.5)	5.9 (5.4-6.8)	**<0.001**
**Arterial blood gas test**						
PaO2, mmHg	65.0 (54.0-78.4)	67.0 (58.0-77.0)	68.8 (59.0-90.0)	63.0 (52.0-78.4)	59.1 (51.0-75.0)	**0.049**
PaCO2, mmHg	43.0 (36.0-55.0)	43.0 (37.0-58.0)	43.0 (38.0-58.0)	44.0 (36.0-55.0)	41.0 (36.0-48.0)	0.52
Lactate, mmol/l	1.10 (0.80-1.80)	1.00 (0.80-1.80)	1.40 (1.00-2.40)	1.00 (0.80-1.50)	1.20 (0.80-1.80)	**0.01**
In-hospital death	45 (18.0 %)	9 (13.2 %)	9 (15.5 %)	8 (14.0 %)	19 (28.4 %)	0.08

*Values are mean ± SD, n (%) or median (25th to 75th percentile)*.

*CSI, congestion score index; ePVS, estimated plasma volume status; COPD, chronic obstructive pulmonary disease; HF, heart failure; ACEi/ARB, angiotensin converting enzyme inhibitor or angiotensin receptor blocker; CPAP, continuous positive airway pressure; BP, blood pressure; CRP, C-reactive peptide; eGFR, estimated glomerular filtration rate; BNP, brain natriuretic peptide*.

*Values in bold style represent significant difference (p < 0.05)*.

### Description of the Population According to CSI and ePVS

When considering subsets of patients divided according to the median of CSI and ePVS (Table 1), high CSI/high ePVS was associated with a frequent use of diuretics prior to admission, poor renal function and low partial pressure of arterial oxygen compared with other CSI/ePVS categories. Similar results were observed across CSI/ePVS subgroups with regard to age, sex, body mass index, comorbidities, congestion signs and BNP.

### Association of CSI/ePVS Interplay With in-hospital Mortality

In-hospital mortality occurred in 18.0% of patients (*N* = 45). By univariable logistic models, neither high admission CSI nor ePVS was associated with a higher risk of in-hospital mortality (all *p*-values > 0.05) (Table 2).

**Table 2:** Logistic regression models for in-hospital mortality.

	**Univariable model**		Multivariable model*	
	**OR (95% CI)**	***P*-value**	**OR (95% CI)**	***P*-value**
**CSI admission variables**				
Continuous CSI (per 0.1 increment)	1.04 (0.98-1.11)	0.19	1.05 (0.95-1.17)	0.36
High CSI vs. Low CSI	1.83 (0.95-3.61)	0.07	1.95 (0.74-5.47)	0.19
**ePVS admission variables**				
Continuous ePVS, ml/g	1.25 (0.99-1.57)	0.54	1.30 (0.92-1.82)	0.13
High ePVS vs. Low ePVS	1.67 (0.87-3.26)	0.13	2.70 (0.99-7.88)	0.06
**CSI and ePVS interplay**				
Low CSI and Low ePVS	(Reference)		(Reference)	
High CSI and Low ePVS	1.20 (0.44-3.31)	0.72	0.90 (0.18-4.63)	0.89
Low CSI and High ePVS	1.07 (0.38-3.00)	0.90	1.30 (0.27-6.64)	0.74
High CSI and High ePVS	2.60 (1.10-6.51)	**0.03**	4.20 (1.10-19.67)	**0.048**
**Interaction test between CSI and ePVS**				
Continuous		**0.03**		
Categorical		0.22		

*Multivariable model included age, male, prior hospitalization for heart failure, prior use of diuretics therapy, use of continuous positive airway pressure, blood urea nitrogen, estimated glomerular filtration rate, brain naturistic peptide, and lactate at admission*.

*OR, odds ratio; CI, confidence interval; CSI, congestion score index; ePVS, estimated plasma volume status. Bold values indicated statistical significance*.

**Figure 2:**
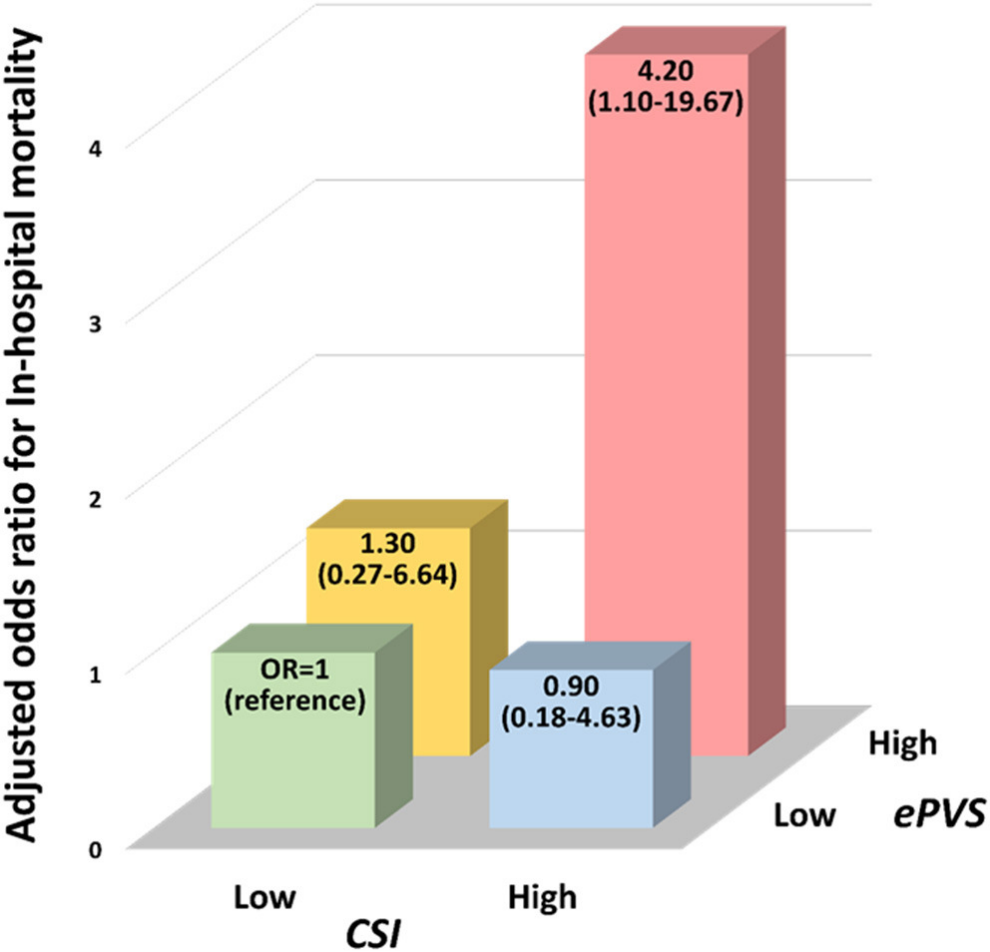
Interplay of congestion score index derived from chest radiography and plasma volume estimated from hemoglobin/hematocrit evaluation with high risk of in-hospital mortality. OR, odds ratio; CSI, congestion score index; ePVS, estimated plasma volume status.

However, a significant interaction between CSI and ePVS was observed for in-hospital mortality (*p*-value for interaction = 0.03). The association of CSI/ePVS-derived subsets with outcome was consequently investigated. In univariable analysis, patients with high CSI/high ePVS were associated with a higher risk of in-hospital mortality [OR [95%CI] = 2.60 [1.10-6.51], *p* = 0.03] compared with those with low CSI/low ePVS. After adjustment for potential clinical covariates including BNP and lactate, patients with high CSI/high ePVS still had a four-fold increase in the risk of in-hospital mortality (adjusted-OR [95%CI] = 4.20 [1.10-19.67], *p* = 0.048) (Figure 2). In contrast, other configurations (either high CSI alone or high ePVS alone) were not significantly associated with in-hospital mortality.

Similarly, the prognostic value of high CSI/ high ePVS was consistent across subgroups of age, sex and comorbidities (coronary artery disease, COPD) or associated diagnosis (pneumonia, pleural effusion) or quality of chest radiogram (*p*-value for interaction > 0.05; Supplementary Table 1).

When considering time-to-event analyses, prognostic value of CSI/ePVS categories for in-hospital mortality appeared to be more prominent after 7 days following ED admission (Supplementary Figure 2). Patients with high CSI/high ePVS tended to be at a higher risk of outcome after 7 days (HR [95%CI] = 6.81 [0.86-53.90], *p* = 0.07), but not before 7 days (HR [95%CI] = 1.16 [0.45-3.01], *p* = 0.76;) (Supplementary Table 2).

### Improvement in Reclassification Associated With in-hospital Mortality

**Figure 3:**
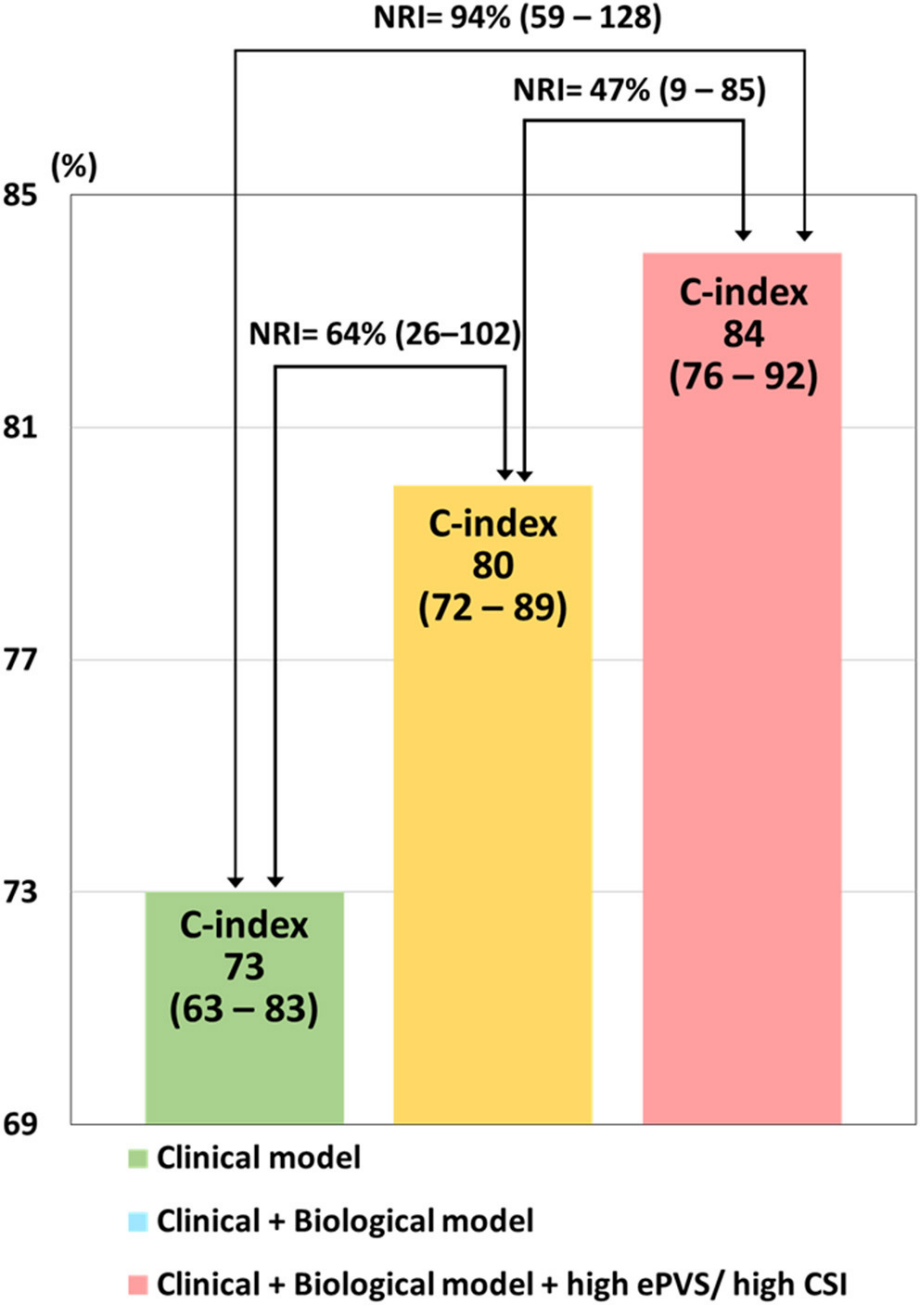
Improvement in reclassification of in-hospital mortality. Clinical model; age, sex, prior admission of heart failure, diuretics therapy and use of continuous positive airway pressure. Biological model; blood urea nitrogen, estimated glomerular filtration rate, brain naturistic peptide, and lactate at admission. NRI, net reclassification improvement; CSI, congestion score index; ePVS, estimated plasma volume status.

Risk prediction of in-hospital mortality as assessed by the clinical model had moderate strength (AUC [95%CI] = 0.73 [0.63-0.83]); the addition of biological variables on top of the clinical model significantly improved reclassification (NRI [95%CI] = 64.0 [26.4-101.6], *p* < 0.001) (Figure 3). Importantly, the addition of high ePVS/high CSI significantly improved reclassification on top of the clinical plus biological models (NRI [95%CI] = 46.9 [8.7-85.1], *p* = 0.02). The combination of clinical, biological models and high ePVS/high CSI resulted in a high level of prediction of the risk of in-hospital mortality (AUC [95%CI] = 0.85 [0.82-0.89]) (Figure 3).

## Discussion

The main findings of the present study are that (1) patients with both high CSI and high ePVS had similar clinical congestion and BNP concentrations than other categories of CSI/ePVS; (2) these patients had a four-fold increased risk of in-hospital mortality, whereas patients with either high CSI or high ePVS alone did not display a poorer prognosis compared with those with low CSI/low ePVS and; (3) the addition of high levels of CSI and ePVS on top of the clinical plus biological prediction models (i.e., age, prior HF admission, renal function, natriuretic peptide, and lactate) was associated with a significant improvement in reclassification for in-hospital mortality. Our results highlight that radiographic pulmonary congestion scoring and PV (estimated from hemoglobin/hematocrit) further refined patient prognosis on top of well-known confounders (i.e., natriuretic peptide and lactate), thus helping emergency physicians in triaging high-risk acute heart failure patients admitted in the emergency department.

### Association of the Combination of CSI and ePVS With in-hospital Mortality

This study included patients with relatively old age (mean age 82 years) and/or a comorbidity burden (i.e., hypertension, chronic obstructive pulmonary disease), which may partly explain a high rate of in-hospital death when compared with other cohorts (23–25). Patients with high CSI/high ePVS had poorer renal function and lower partial pressure of arterial oxygen, which may express a more severe congestion status in these patients (2, 26, 27). However, no difference in clinical congestion (i.e., rales and leg edema) and natriuretic peptides was observed across CSI/ePVS categories, suggesting that CSI/ePVS categorization goes beyond the routine assessment of congestion markers (i.e., clinical exam and natriuretic peptides quantification). Intriguingly, we observed a poor correlation between CSI and ePVS, which may be partly explained by the fact that not only fluid accumulation - as expressed by PV- but also fluid redistribution from splanchnic venous system may contribute to degrees of pulmonary congestion (3, 28–30). Different interplays of CSI and ePVS may be related to different pathophysiological scenarios.

Our results showed that only the simultaneous presence of pulmonary and intravascular congestion (namely high CSI/high ePVS) conferred a higher risk of in-hospital mortality, whereas patients with only one severe congestion (pulmonary congestion or intravascular congestion) marker had a comparable risk of adverse outcome when compared with those without. In previously published data, a uniform definition of congestion not acknowledging the distinction between pulmonary and intravascular congestion provided a limited predictive value of congestion for in-hospital mortality (6, 7). Our results, therefore, suggest that the initial assessment of pulmonary congestion in the ED, based on CSI sensitized by ePVS, can provide better a risk-stratification in patients admitted for AHF. Furthermore, worse prognosis of high CSI/ high ePVS was likely observed after 7 days of ED admission, suggesting it may not be a marker of an immediate risk.

### Discriminative Value of CSI and ePVS Interplay on in-hospital Mortality

The management of AHF in the ED limits available tools of assessing the severity of congestion. Several prognostic markers (i.e., renal function, natriuretic peptide, and lactate) are widely used for triage assessment, allowing a determinant of decision-making in patients admitted for AHF in the ED (31, 32). However, these features are not specific for pulmonary congestion, which eventually causes acute dyspnea in HF. Importantly, in the present study, we demonstrate the incremental value of CSI/ePVS interplay in predicting prognosis on top of well-known prognostic markers, suggesting the need for the plausible assessment of congestion associated with the occurrence of dyspnea in patients admitted for AHF.

### Clinical Perspectives

More than 80% of AHF patients expressing dyspnea initially present to the ED (33, 34). Current recommendations advocate for initiating AHF treatment including diuretic treatment within an hour of admission, which may potentially improve in-hospital outcome (35, 36). However, congestion assessment tools (e.g., echocardiogram and lung ultrasound) may not be available to emergency physicians in routine clinical practice (25). Therefore, our readily and widely available method of assessing congestion, based on chest radiography and hemoglobin/hematocrit, may provide a better identification of patients at markedly high-risk of ED admission. An adequately powered prospective study is further needed to confirm its prognostic value for in-hospital mortality in the ED.

### Limitations

The results should be interpreted in light of the following limitations. First, this is a retrospective single-center cohort study including a moderate number of relatively old patients (mean age 82 years) with a high level of comorbidity burdens, leading to limited statistical power and possible residual confounding. Particularly, prognostic value of ePVS/CSI interplay was corroborated by results in logistic regression model, but not in time-to-event analyses in which high CSI/ high ePVS subgroup had a nominal *p*-value of 0.07 for high risk of outcome. Further research with a larger sample size is needed. Second, CSI is a semi-quantitative tool with some subjective assessment. However, in our experience, a short training period of ~3 h is sufficient to achieve accurate and reproducible scoring. The prognostic value of CSI/ePVS, in addition, was not significantly influenced by the quality of the chest radiographic images, suggesting that our findings may apply to a broad spectrum of clinical settings. Third, chest radiogram was generally assessed as soon as possible after ED admission, but the exact time at which chest radiogram was performed is lacking. We, thus, cannot ascertain that initial therapeutic interventions (i.e., furosemide, continuous positive airway pressure) may not impact CSI evaluation, although this would be the case in routine ED practice in most centers. Fourth, in addition to lack of direct measured PV, there is no accepted normal value of ePVS; however, PV estimated from hemoglobin/hematocrit had a good correlation with measured PV in a recent report (37), and ePVS > 5.5 g/dl may be a useful threshold to identify congestion in HF based on previously published data (13, 14, 38). Fifth, New York Heart Association functional class and echocardiographic data were not available, which could potentially have modified the prognostic value of CSI/ePVS in adjusted models (9). However, echocardiogram is currently scarcely available to screen high-risk patients in the ED in most countries (35, 39).

## Conclusion

In relatively old (mean age 82 years) patients hospitalized for acute HF with a high level of comorbidity burdens, pulmonary/intravascular congestion scoring categories as assessed by CXR and ePVS had similar severity of congestion assessed by physical examination/natriuretic peptides. However, high CSI and high ePVS identified patients at a high-risk of in-hospital death, improving patient-risk stratification beyond clinical plus biological prognostic models. These results suggest that this readily and widely available congestion assessment, integrating chest radiography and hemoglobin/hematocrit, may represent a valuable triage tool for patients admitted for AHF with dyspnea in the ED.

## Data Availability Statement

The raw data supporting the conclusions of this article will be made available by the authors, without undue reservation.

## Ethics Statement

The studies involving human participants were reviewed and approved by CNIL (Number R2016-08). Written informed consent for participation was not required for this study in accordance with the national legislation and the institutional requirements.

## Author Contributions

MK and AD drafted the manuscript. NG supervised the manuscript. All authors edited the paper and/or provided constructive comments.

## Funding

MK was granted by the RHU Fight-HF, a public grant overseen by the French National Research Agency (ANR) as part of the second “Investissements d'Avenir” program (ANR-15RHUS-0004). MK, PR, KD, NG, and FZ are supported by the RHU Fight-HF, a public grant overseen by the French National Research Agency (ANR) as part of the second “Investissements d'Avenir” program (ANR-15-RHUS-0004) and by the FrenchPIA project “Lorraine Universite' d'Excellence” (ANR-15-IDEX-04-LUE).

## Conflict of Interest

NG receives honoraria from Novartis and Boehringer. TC receives fees from Novartis for scientific board. FZ and PR are the cofounders of CardioRenal. FZ reports personal fees from Boehringer Ingelheim, Janssen, Novartis, Boston Scientific, Amgen, CVRx, AstraZeneca, Vifor Fresenius, Cardior, Cereno pharmaceutical, Applied Therapeutics, Merck, Bayer and Cellprothera, and is a founder of Cardiovascular Clinical Trialists. PR reports grants and personal fees from AstraZeneca, Bayer, CVRx, Fresenius, and Novartis, personal fees from Grunenthal, Servier, Stealth Peptides, Vifor Fresenius Medical Care Renal Pharma, Idorsia, NovoNordisk, Ablative Solutions, G3P, Corvidia and Relypsa. The remaining authors declare that the research was conducted in the absence of any commercial or financial relationships that could be construed as a potential conflict of interest.

## Publisher's Note

All claims expressed in this article are solely those of the authors and do not necessarily represent those of their affiliated organizations, or those of the publisher, the editors and the reviewers. Any product that may be evaluated in this article, or claim that may be made by its manufacturer, is not guaranteed or endorsed by the publisher.
