# Supplementary material for: A Combination of Chest Radiography and Estimated Plasma Volume May Predict In-Hospital Mortality in Acute Heart Failure
Source: Front Cardiovasc Med. 2022 Jan 11;8:752915. doi: 10.3389/fcvm.2021.752915 (PMC8787280; doi:10.3389/fcvm.2021.752915)
Supplement: Supplementary file 1 [file Data_Sheet_1.docx]

**
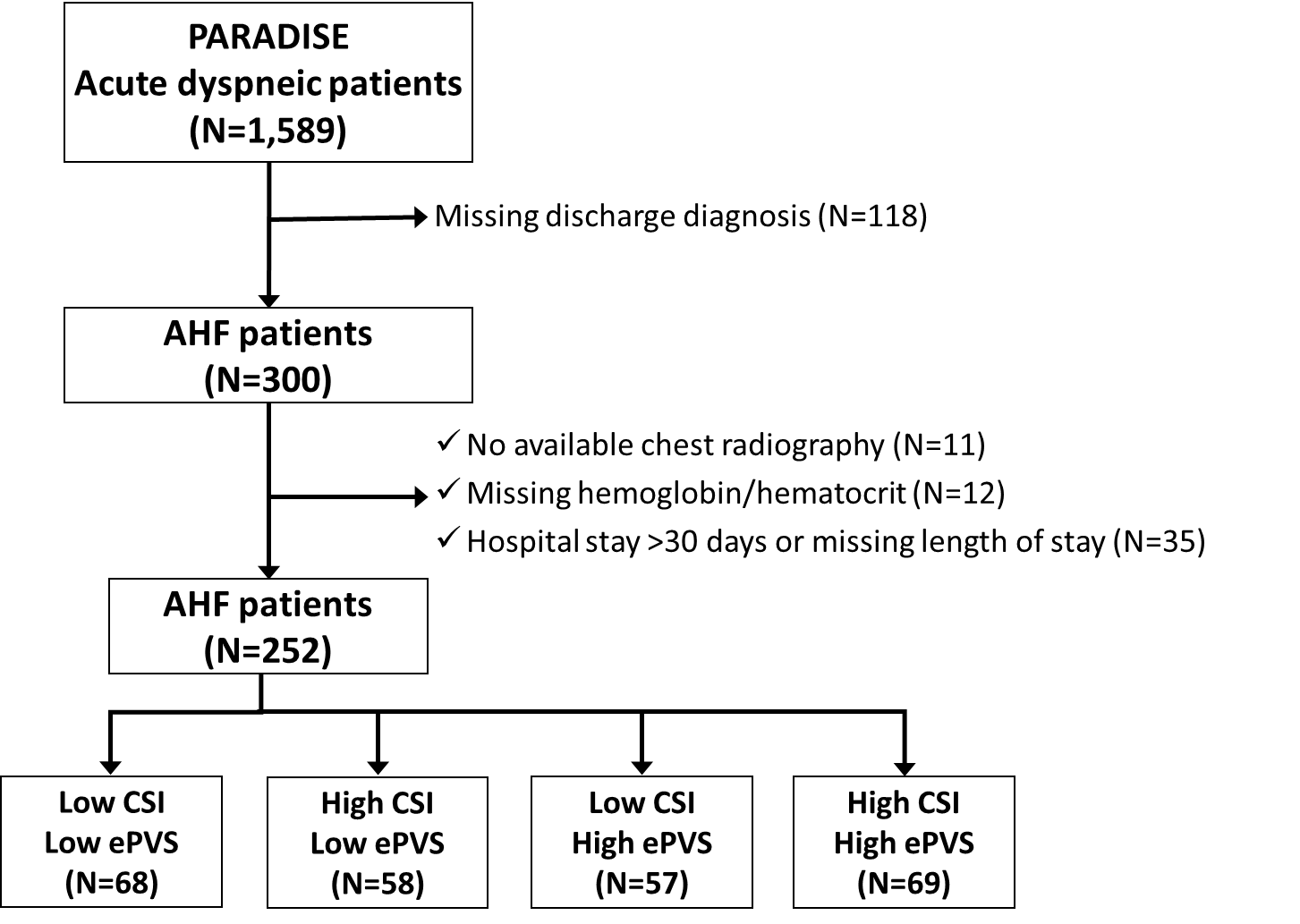
Supplementary figure 1. Study flow chart**

**Supplementary figure 2. Survival curve for the inhospital mortality according to ePVS/CSI categories**


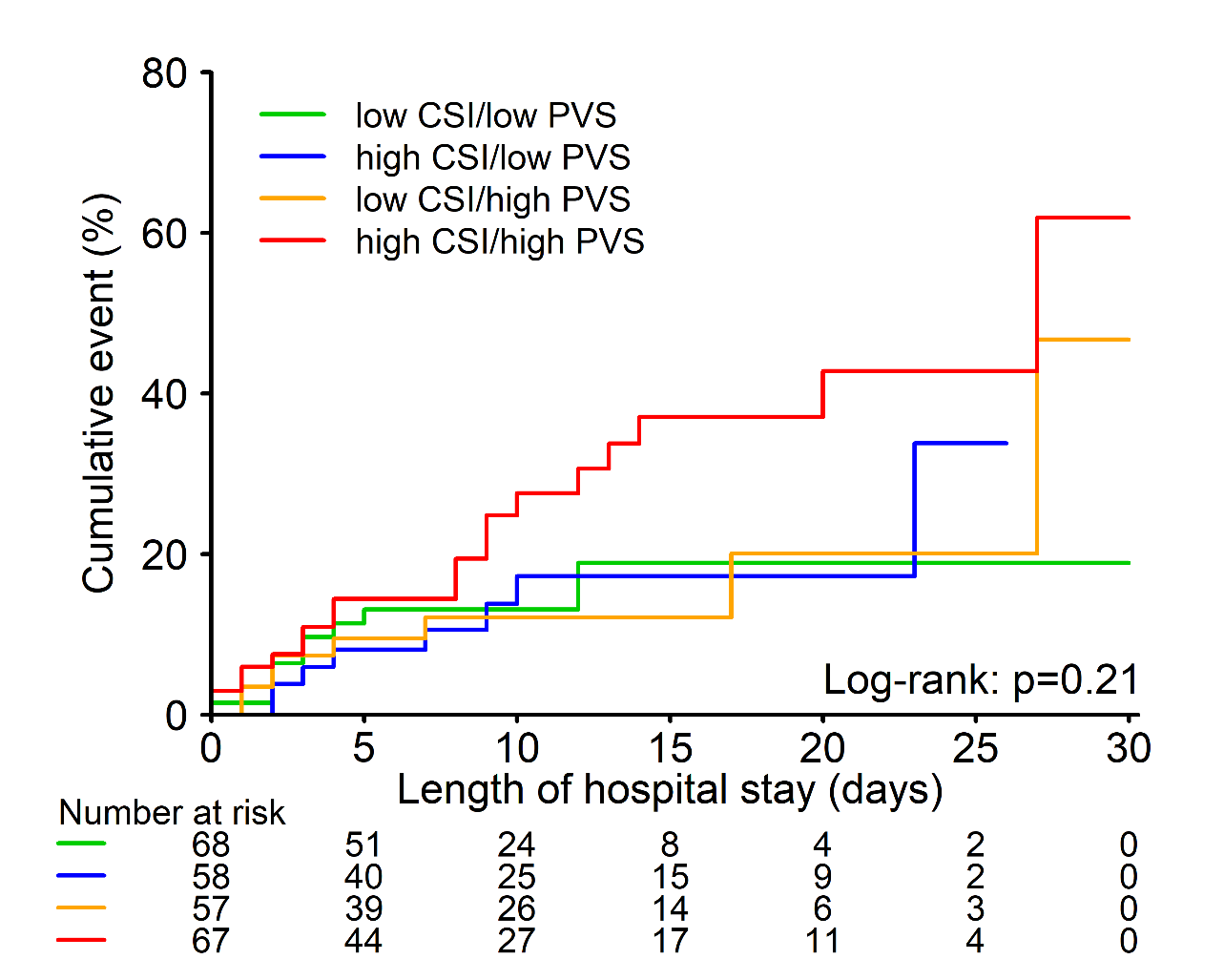


**Supplementary table 1. Interaction between CSI/ePVS and baseline factors on in-hospital mortality**

|  | **p-value for interaction** |
| --- | --- |
| **Age >80 years** | 0.98 |
| **Sex** | 0.09 |
| **Hypertension** | 0.21 |
| **Coronary artery disease** | 0.78 |
| **COPD** | 0.45 |
| **Pneumonia** | 0.88 |
| **Pleural effusion in chest radiogram** | 0.18 |
| **Quality of chest radiogram** | 0.07 |

**Supplementary table 2. Cox proportional hazard model for in-hospital mortality before and after 7 days following emergency department admission**

|  | <7 days | | | ≥7 days | | |
| --- | --- | --- | --- | --- | --- | --- |
|  | **HR** | **95% CI** | **p-value** | **HR** | **95% CI** | **p-value** |
| CSI continuous (per 0.1) | 0.99 | 0.99-1.24 | 0.92 | 1.11 | 0.99-1.24 | 0.07 |
| High CSI vs Low CSI | 1.01 | 0.48-2.12 | 0.98 | 4.30 | 1.23-15.10 | **0.02** |
| ePVS continuous | 0.94 | 0.70-1.26 | 0.68 | 1.40 | 1.04-1.88 | **0.03** |
| High ePVS vs Low ePVS | 1.18 | 0.56-2.49 | 0.66 | 1.80 | 0.62-5.21 | 0.28 |
| Low CSI & Low ePVS |  | (reference) |  |  | (reference) |  |
| High CSI & Low ePVS | 0.74 | 0.24-2.27 | 0.60 | 3.75 | 0.14-16.97 | 0.24 |
| Low CSI & High ePVS | 0.90 | 0.31-2.61 | 0.85 | 1.52 | 0.41-33.93 | 0.73 |
| High CSI & High ePVS | 1.16 | 0.45-3.01 | 0.76 | 6.81 | 0.86-53.90 | 0.07 |
